# A Theoretical Study on the Role of Astrocytic Activity in Neuronal Hyperexcitability by a Novel Neuron-Glia Mass Model

**DOI:** 10.1186/s13408-016-0042-0

**Published:** 2016-12-21

**Authors:** Aurélie Garnier, Alexandre Vidal, Habib Benali

**Affiliations:** 1 Laboratoire d’Imagerie Biomédicale (LIB), Sorbonne Universités, UPMC Univ Paris 06, CNRS, INSERM, Paris, 75013 France; 2 Laboratoire de Mathématiques et Modélisation d’Évry (LaMME), CNRS UMR 8071, Université d’Évry-Val-d’Essonne, Évry, 91000 France

**Keywords:** ODE model in neuroscience, Qualitative analysis of dynamical systems, Bifurcations, Neuron-astrocyte interactions, GABAergic and glutamatergic neurotransmissions, Excitability modulation, Neuronal hyperexcitability

## Abstract

Recent experimental evidence on the clustering of glutamate and GABA transporters on astrocytic processes surrounding synaptic terminals pose the question of the functional relevance of the astrocytes in the regulation of neural activity. In this perspective, we introduce a new computational model that embeds recent findings on neuron–astrocyte coupling at the mesoscopic scale intra- and inter-layer local neural circuits. The model consists of a mass model for the neural compartment and an astrocyte compartment which controls dynamics of extracellular glutamate and GABA concentrations. By arguments based on bifurcation theory, we use the model to study the impact of deficiency of astrocytic glutamate and GABA uptakes on neural activity. While deficient astrocytic GABA uptake naturally results in increased neuronal inhibition, which in turn results in a decreased neuronal firing, deficient glutamate uptake by astrocytes may either decrease or increase neuronal firing either transiently or permanently. Given the relevance of neuronal hyperexcitability (or lack thereof) in the brain pathophysiology, we provide biophysical conditions for the onset identifying different physiologically relevant regimes of operation for astrocytic uptake transporters.

## Introduction

Neural activity in the brain results from an interplay between neuronal excitation and inhibition and is subjected to the supply of nutrients by the cerebral blood flow. At the subcellular (microscopic) scale, neural activity propagates through axons and, mostly, chemical synapses which transmit electrical signals from one (presynaptic) neuron to another (postsynaptic) one, by release of neurotransmitters, like glutamate and GABA for example, in the extracellular space (or synaptic cleft) between pre- and postsynaptic terminals [1, 2]. These neurotransmitters diffuse in the synaptic cleft, binding and activating postsynaptic receptors, which can regulate the depolarization state of the postsynaptic neuron, triggering firing thereof. In parallel, spillout of these neurotransmitters from the synaptic cleft to the perisynaptic extracellular space may also activate receptors and transporters on perisynaptic astrocytic processes, with the potential to trigger glutamate release by the latter glutamate into the extra-synaptic space [3, 4].

The predominant expression of high affinity astrocytic glutamate and GABA transporters in the proximity of synaptic terminals [5–7] raises the question of the functional relevance of the astrocytic uptake in the regulation of synaptic transmission and thus in the tone of neural excitability in the brain pathophysiology [8–11]. In this perspective, computational modeling provides a key tool to aid diagnostic and possible treatment of brain pathophysiology, providing a rationale to interpret electrophysiological data when, at the current state of technology, dynamical recordings of intracranial electroencephalography (EEG) cannot provide any information on underlying concentrations of neurotransmitters. Over the past decade, modeling efforts made in this regard have exploited several directions. There exists indeed a wealth of published models for neuron–astrocyte interactions which consider neurotransmitter and ion dynamics at different scales: subcellular [12–16], cellular [4, 17–19] and supracellular, including a hemodynamic compartment [20–22], and network [23, 24]. Despite the different scales of analysis, consensus has grown from these studies as to the existence of a plausible causal link between astrocyte activation and neuronal (hyper)excitability [13, 15, 16, 23, 25]. With few exceptions [4, 16, 17], however, a main limitation of these models is that they are hardly mathematically tractable and the computational demand for scaling them up to meso/macroscopic scales requires dedicated computing platforms. A possible alternative which brings together computational portability with mathematical tractability is represented by neuron–astrocyte mass modeling of dynamical interactions between neurons and astrocytes populations. Indeed the mass approach is well suited to reproduce mesoscopic data obtained by current EEG technology [26]. The aggregated yet biologically significant parameters of a mass model can in fact efficiently be chosen so as to fit experimental data [27]. Moreover, such models are usually low dimensional and thus amenable to analytical treatment aimed at interpreting experimental observations as well as identifying parameter sets for different dynamical regimes of the model [28, 29]. In particular, we benefit from the bifurcation analysis of the Neural Mass Model (NMM) performed in [29] to characterize in this study, the impact of astrocyte on neuronal activity. More exactly, our focus for the present study is on Noise Induced Spiking (NIS), which is characterized by quiescent phases of neuronal activity separated by isolated spikes resulting from synchronous neuronal activations. NIS typically mimics local field potentials (LFP) recordings obtained during pre-ictal phases of epilepsy as shown in [29] and may thus be used as a predictor for the insurgence of neuronal hyperexcitability.

A novel computational model based on the neural mass approach that focuses on astrocyte dynamics at the mesoscopic scale has recently been proposed [30]. This model links the LFP signal representative of neural activity measured by intracranial EEG to the cerebral blood flow dynamics measured by laser doppler recordings of astrocyte activity. Although this model incorporates astrocytic recycling of glutamate and GABA it does not take into account the modulation of neuronal excitability by extracellular neurotransmitter concentrations. In the following, we present an extension of this model that includes such modulation in the context of bilateral neuron–astrocyte interactions. In particular, we pursue an analytical characterization of the impact of the key model parameters of astrocytic activity on neuronal hyperexcitability.

Analytical investigation of our model allows us to make several predictions on the effect of deficiencies of GABA and glutamate astrocytic uptake on neural activity. In particular, we identify three possible neural regimes in the presence of deficiency of astrocytic glutamate uptake, respectively, consistent with reduced activity, transient or permanent hyperexcitability. Such a spectrum of responses is substantiated by the analysis of the dynamical structure of the model, which allows us to derive explicit conditions on the parameters involved in the astrocytic feedback corresponding to each type of regime and put emphasis on the delicate balance between neuronal excitation and inhibition and its sensitivity to extracellular concentrations of neurotransmitters.

## Bilaterally Coupled Neuron–Astrocyte Mass Modeling

**Fig. 1:**
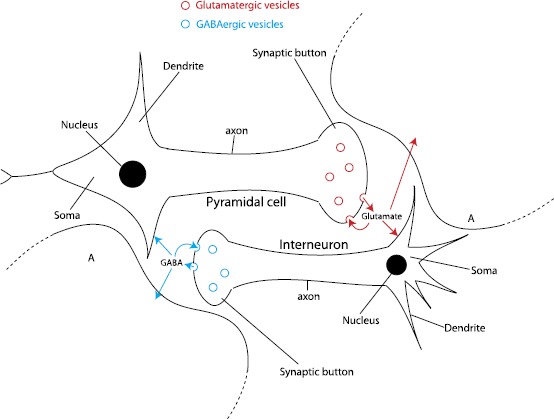
Scheme of neurotransmission mechanisms and neurotransmitter uptake. *Red circles*: glutamatergic vesicles. *Blue circles*: GABAergic vesicles. *Red arrows*: exchanges of glutamate. *Blue arrows*: exchanges of GABA. A: astrocytes

Physiologically, a pyramidal neuron (resp. an interneuron) releases glutamate (resp. GABA) in the synaptic cleft from where it binds to receptors on the postsynaptic neuron, which stands for the main mechanism of synaptic transmission. In the NMM, the role of neurotransmitter fluxes in this mechanism is implicit since the neuronal activity is reproduced at the scale of populations. Henceforth, the tonic impacts of neurotransmitters (GABA and glutamate) are embedded in conversion processes from average pulse density into postsynaptic potentials. Uptake processes of the neurotransmitters by the local astrocyte and presynaptic neuron regulates their concentration in the extracellular space (Fig. 1). In the presynaptic neuron, the uptake completes the stock whereas the uptake by the astrocyte triggers a cascade of reactions linked with the modulation of synaptic transmission (differentially according to the type of neurotransmitters) and the hemodynamics. This indirect mechanism involves concentrations represented by explicit variables of the glial compartment of our neuron–astrocyte mass model. Hence, feedbacks from the glial compartment onto the neural dynamics stand for the modulations of excitability (or more precisely the activation threshold of each population) by the extracellular concentration of each neurotransmitters.

In the following, we set to describe our mass modeling approach to account for the effect of extracellular glutamate and GABA concentrations on neural activity. With this aim, we start by recalling the NMM setup originally studied in [29, 30], its properties and time-series pattern that it produces. We then introduce our modeling arguments to develop bilateral coupling between the astrocytic and neuronal compartments of the model. Finally, we leverage on the novel dynamical features brought forth by inclusion of this bilateral coupling with respect to the original model in [29, 30].

### Neural Mass Model

**Fig. 2:**
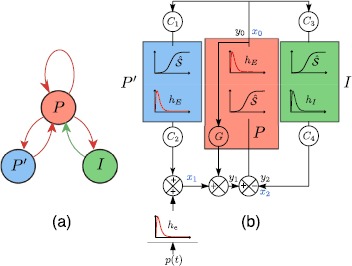
Two schematic representations of the NMM with double excitatory feedbacks. *P*: main population of pyramidal neurons. *I*: interneuron population. $P'$: secondary population of pyramidal neurons. *Red* (resp. *green*) *arrows* in (**a**): excitatory (resp. inhibitory) interactions. *Box* $h_{E}$ (resp. $h_{I}$): second-order process converting action potentials into excitatory (resp. inhibitory) postsynaptic potential. *Box* $\hat{\mathcal {S}}$: process converting average membrane potential into average action potential density discharge by neurons of populations *P*, $P'$, and *I*, respectively. $C_{i}$ for $1 \leq i \leq4$: coupling gain parameters depending on the maximum number *C* of synaptic connections between two populations. *G*: direct feedback coupling gain. $p(t)$: external input. $y_{0}$, $y_{1}$, $y_{2}$: state variables. $x_{0}$, $x_{1}$, $x_{2}$: intermediary variables

The NMM represents the dynamical interactions between two neural populations at a mesoscopic scale: a main population of pyramidal neurons (*P*) and a population of inhibitory interneurons (*I*). For the excitatory feedback of pyramidal neurons, two approaches have been considered in the literature: a direct link from their output to their input and an indirect track through synaptic coupling with distant neurons. The latter way of modeling amounts to considering an intermediary population of pyramidal neurons $P'$ interacting with *P* through synaptic connections. Both direct and indirect approaches model the excitatory synaptic interaction between neighbor principal cells, usually named collateral excitation. From the modeling perspective, we cannot privilege one type of feedback over the other, since both these couplings are physiologically relevant and can co-exist, a very local one and a more or less distant one. The NMM used in this article, proposed and studied in [29], includes both feedback circuits. Hence, it includes three feedback loops on population *P* activity: an inhibitory feedback through the interneuron population *I*, a direct excitatory feedback of *P* onto itself (referred to as “direct feedback”) and an indirect excitatory feedback (referred to as “indirect feedback”) involving the population $P'$ (Fig. 2(a)).

The conversion process of average pulse density into excitatory and inhibitory postsynaptic potential, respectively, are based on the following *α*-functions introduced by Van Rotterdam *et al.* [31]:

$$\begin{aligned} h_{E}(t) =& A a t e^{-a t}, \\ h_{I}(t) =& B b t e^{-b t}. \end{aligned}$$

These are fundamental solutions (also known as Green’s functions) for the differential operators ${\mathcal{F}}_{E}$ and ${\mathcal{F}}_{I}$, respectively, defined by

1a $$\begin{aligned} {\mathcal{F}}_{E}(h_{E}) =& \frac{1}{A} \biggl( \frac{1}{a} \frac{d^{2} h_{E}}{dt^{2}}+2 h_{E}'+a h_{E} \biggr), \end{aligned}$$

1b $$\begin{aligned} {\mathcal{F}}_{I}(h_{I}) =& \frac{1}{B} \biggl( \frac{1}{b} \frac{d^{2} h_{I}}{dt^{2}}+2 h_{I}'+b h_{I} \biggr). \end{aligned}$$

In this framework, the parameter *A* (resp. *B*) stands for the average excitatory (resp. inhibitory) synaptic gain and tunes the amplitude of excitatory (resp. inhibitory) postsynaptic potentials. Additionally, $\frac{1}{a}$ (resp. $\frac{1}{b}$) represents the time constant of excitatory (resp. inhibitory) postsynaptic potentials representative of the kinetics of synaptic connections and delays introduced by circuitry of the dendritic tree [31–33]. Following Freeman’s work [32], the functions converting the average membrane potential into an average pulse density can be approximated by sigmoids of the type

$${\mathcal{S}}(x,x_{{\mathrm{th}}},r_{{\mathrm{sl}}}) = \frac{1}{1+e^{r_{{\mathrm{sl}}} (x_{{\mathrm{th}}}-x)}}. $$

Yet, for the sake of compactness of the NMM presentation, we introduce an auxiliary parameterization after [32]:

$$\hat{\mathcal{S}}(x,v) = 2 e_{0} {\mathcal{S}}(x,v,r) = \frac{2 e_{0}}{1+e^{r (v-x)}}, $$

where $2 e_{0}$ represents the maximum discharge rate, *v* the excitability threshold and *r* the sigmoid slope at the inflection point. Finally, the NMM receives an excitatory input $p(t)$ standing for the action on population *P* of neural populations in other areas through long-range synaptic connections. This input can be either deterministic or stochastic, and in this case, being modeled for example by a Gaussian process.

Now we can write the dynamics for the intermediary variables $x_{0}$, $x_{1}$ and $x_{2}$, which represent the outputs of the population *P*, the sum of population $P'$ output and the external input $p(t)$, and the population *I*, respectively (Fig. 2(b)):

2a $$\begin{aligned} \frac{d^{2} x_{0}}{dt^{2}} =& A a \hat{\mathcal{S}}(x_{1}+G x_{0}-x_{2},v_{P})-2 a \frac{d x_{0}}{dt}-a^{2} x_{0}, \end{aligned}$$

2b $$\begin{aligned} \frac{d^{2} x_{1}}{dt^{2}} =& A a C_{2} \hat{\mathcal{S}}(C_{1} x_{0},v_{P'})-2 a \frac{d x_{1}}{dt}-a^{2} x_{1}+A a p(t), \end{aligned}$$

2c $$\begin{aligned} \frac{d^{2} x_{2}}{dt^{2}} =& B b C_{4} \hat{\mathcal{S}}(C_{3} x_{0},v_{I})-2 b \frac{d x_{2}}{dt}-b^{2} x_{2}. \end{aligned}$$

Parameters $C_{i}$ ($i\in \{1,2,3,4\}$) represent the average number of synapses between two populations. Following [34], each $C_{i}$ is proportional to the maximum number *C* of synapses between two populations. The excitation of *P* by its own output, resulting from the intra-population synaptic connections, is weighted by the coupling gain *G*.

For the sake of comparison, we use a variable change to obtain the same state variables as in the Jansen–Rit model [35]: the excitatory output ($y_{0}=x_{0}$) and the excitatory ($y_{1}=x_{1}+G x_{0}$) and inhibitory ($y_{2}=x_{2}$) inputs of the main population *P*. The output $y_{0}$ acts on the secondary pyramidal neuron population $P'$ and on the interneuron population *I*. To analyze the model, we write the dynamics of the state variables $y_{0}$, $y_{1}$, and $y_{2}$ as a system of first order differential equations:

3a $$\begin{aligned} \frac{d y_{0}}{dt} =& y_{3}, \end{aligned}$$

3b $$\begin{aligned} \frac{d y_{1}}{dt} =& y_{4}, \end{aligned}$$

3c $$\begin{aligned} \frac{d y_{2}}{dt} =& y_{5}, \end{aligned}$$

3d $$\begin{aligned} \frac{d y_{3}}{dt} =& A a \hat{\mathcal{S}}(y_{1}-y_{2},v_{P})-2 a y_{3}-a^{2} y_{0} , \end{aligned}$$

3e $$\begin{aligned} \frac{d y_{4}}{dt} =& A a C_{2} \hat{\mathcal{S}}(C_{1} y_{0},v_{P'})+A a G \hat{\mathcal{S}}(y_{1}-y_{2},v_{P}) \\ &{} -2 a y_{4}-a^{2} y_{1}+A a p(t) , \end{aligned}$$

3f $$\begin{aligned} \frac{d y_{5}}{dt} =& B b C_{4} \hat{\mathcal{S}}(C_{3} y_{0},v_{I})-2 b y_{5}-b^{2} y_{2}. \end{aligned}$$

In this study, we consider the local field potential $\operatorname{LFP}(t)=y_{1}(t)-y_{2}(t)$ [33] as the main output of the model. It is important to note that, generally, studies of neural mass models, such as Jansen–Rit model, only considered the case with the same constant excitability thresholds for all populations, *i.e.*

$$v_{P}=v_{P'}=v_{I}=v_{0}. $$

### Astrocyte Model: Glutamate and GABA Concentration Dynamics

**Fig. 3:**
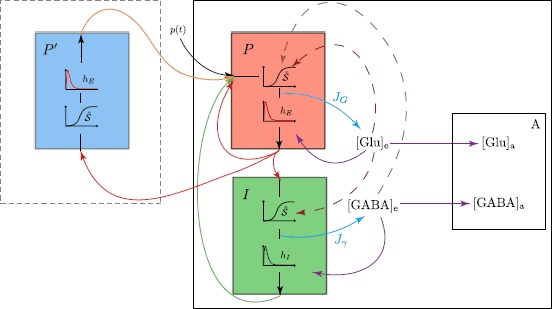
Neuron-astrocyte model with astrocyte feedback. *P* and $P'$: main and secondary populations of pyramidal neurons. *I*: interneuron population. $p(t)$: external input on population *P*. ${[\mathrm{Glu}]_{\mathrm{e}}}$ and ${[\mathrm{GABA}]_{\mathrm{e}}}$: glutamate and GABA extracellular concentrations. ${[\mathrm{Glu}]_{\mathrm{a}}}$ and ${[\mathrm{GABA}]_{\mathrm{a}}}$: glutamate and GABA astrocyte concentrations. *Red arrows*: $P \rightarrow P$, $P \rightarrow I$ and $P \rightarrow P'$ couplings. *Orange arrow*: $P' \rightarrow P$ coupling. *Green arrow*: $I \rightarrow P$ coupling. *Cyan arrows*: glutamate and GABA release by populations *P* and *I* into extracellular space (fluxes $J_{\gamma }^{en}$ and $J_{\gamma}$). *Purple arrows*: astrocyte and neural uptakes of neurotransmitters. *Red dashed arrows*: glutamate feedbacks on populations *P* and *I*. *Brown dashed arrow*: GABA feedback on population *P*

For reproducing the astrocyte activity, we use the model introduced in [30]. It focuses on the dynamics of glutamate and GABA concentrations, which are the main neurotransmitters of the central nervous system. In [30], the neuron–astrocyte coupling is feedforward: the astrocyte dynamics is driven by the neural activity, generated by the Jansen–Rit model, but it does not impact the neural compartment. The model considers the dynamics of glutamate and GABA concentrations, locally to the main population *P* of pyramidal neurons, at different stages of the recycling mechanism. The local nature of this interaction implies that the firing rate of the secondary population $P'$ of pyramidal neurons does not impact the astrocyte dynamics associated with the neighboring astrocytes of the main population *P*. The mechanism is as follows (Fig. 3): excited pyramidal neurons (resp. interneurons) release glutamate (resp. GABA) in the extracellular space (synaptic cleft). Astrocytes and presynaptic neurons uptake the neurotransmitters. Astrocytes recycle or consume the neurotransmitters, while the presynaptic neurons capture them to complete their stock.

Following [30] the astrocyte compartment is built on the firing rate of the pyramidal neuron population and the firing rate of the interneuron population. The state variables are (i) $J_{G}$ and $J_{\gamma}$: the fluxes of glutamate and GABA from neurons to extracellular space, (ii) ${[\mathrm{Glu}]_{\mathrm{e}}}$ and ${[\mathrm{GABA}]_{\mathrm{e}}}$: the neurotransmitter concentrations in the extracellular space, (iii) ${[\mathrm{Glu}]_{\mathrm{a}}}$ and ${[\mathrm{GABA}]_{\mathrm{a}}}$: the quantity of neurotransmitters recycled and consumed by the astrocytes. The dynamics governing $J_{G}$ and $J_{\gamma}$ is driven by second-order differential operators similar to those for synaptic transfer dynamics introduced in (1a)-(1b) [31, 36], *i.e.*:

$$\begin{aligned} {\mathcal{F}}_{G}(h_{G}) =& \frac{1}{W} \biggl( \frac{1}{w_{1}} \frac{d^{2} h_{G}}{dt^{2}}+\frac{w_{1}+w_{2}}{w_{1}} \frac{d h_{G}}{dt}+w_{2} h_{G} \biggr), \\ {\mathcal{F}}_{\gamma}(h_{\gamma}) =& \frac{1}{Z} \biggl( \frac {1}{z_{1}} \frac{d^{2} h_{\gamma}}{dt^{2}}+\frac{z_{1}+z_{2}}{z_{1}} \frac{d h_{\gamma}}{dt}+z_{2} h_{\gamma} \biggr). \end{aligned}$$

Like for the synaptic transfer functions in (1a)-(1b), the parameter *W* (resp. *Z*) tunes the peak amplitude of glutamate (resp. GABA) concentrations and the parameters $w_{1}$ and $w_{2}$ (resp. $z_{1}$ and $z_{2}$) tune the rise and decay times of glutamate (resp. GABA) release transfer function. This dynamics is well suited to reproduce qualitative and quantitative properties of rise and decay of neurotransmitter concentrations as shown in [30].

Astrocytic uptake of glutamate and GABA from the extracellular space may be modeled by Michaelis–Menten kinetics [30], using the following function:

4 $$ {\mathcal{H}}(x,k) = \frac{x}{x+k}. $$

The dynamics of the extracellular concentrations (${[\mathrm{Glu}]_{\mathrm{e}}}$ and ${[\mathrm{GABA}]_{\mathrm{e}}}$) result from neurotransmitter fluxes $J_{G}$ and $J_{\gamma}$ originating from synaptic release fluxes as just described. Intracellular concentrations of glutamate and GABA in the astrocyte instead mirror uptake dynamics and a nonspecific first order (linear) kinetics degradation [27].

To summarize, the feedforward model obtained by coupling the neural mass model defined by (3a)-(3f) with astrocytic dynamics introduced in [30] reads

5a $$\begin{aligned} \frac{d^{2} y_{0}}{dt^{2}} =& A a \hat{\mathcal{S}}(y_{1}-y_{2},v_{P})-2 a \frac{d y_{0}}{dt}-a^{2} y_{0}, \end{aligned}$$

5b $$\begin{aligned} \frac{d^{2} y_{1}}{dt^{2}} =& A a C_{2} \hat{\mathcal{S}}(C_{1} y_{0},v_{P'})+A a G \hat{\mathcal{S}}(y_{1}-y_{2},v_{P}) \\ &{} -2 a \frac{d y_{1}}{dt}-a^{2} y_{1}+A a p(t), \end{aligned}$$

5c $$\begin{aligned} \frac{d^{2} y_{2}}{dt^{2}} =& B b C_{4} \hat{\mathcal{S}}(C_{3} y_{0},v_{I})-2 b \frac{d y_{2}}{dt}-b^{2} y_{2}, \end{aligned}$$

5d $$\begin{aligned} \frac{d^{2} J_{G}}{dt^{2}} =& W w_{1} \hat{\mathcal {S}}(y_{1}-y_{2},v_{P})-(w_{1}+w_{2}) \frac{d J_{G}}{dt} -w_{1} w_{2} J_{G}, \end{aligned}$$

5e $$\begin{aligned} \frac{d {[\mathrm{Glu}]_{\mathrm{e}}}}{dt} =& J_{G}-\bigl(V_{G}^{{\mathrm{ae}}} + V_{G}^{{\mathrm{ne}}}\bigr) {\mathcal{S}}\bigl({[\mathrm{Glu}]_{\mathrm{e}}}, s_{g}, r_{g}\bigr), \end{aligned}$$

5f $$\begin{aligned} \frac{d {[\mathrm{Glu}]_{\mathrm{a}}}}{dt} =& V_{G}^{{\mathrm{ae}}} {\mathcal{S}}\bigl({[ \mathrm{Glu}]_{\mathrm{e}}}, s_{g}, r_{g} \bigr)-V_{G}^{c} {[\mathrm{Glu}]_{\mathrm{a}}}, \end{aligned}$$

5g $$\begin{aligned} \frac{d^{2} J_{\gamma}}{dt^{2}} =& Z z_{1} \hat{\mathcal{S}}(C_{3} y_{0},v_{I})-(z_{1}+z_{2}) \frac{d J_{\gamma}}{dt} -z_{1} z_{2} J_{\gamma}, \end{aligned}$$

5h $$\begin{aligned} \frac{d {[\mathrm{GABA}]_{\mathrm{e}}}}{dt} =& J_{\gamma}-V_{\gamma}^{{\mathrm{ae}}} { \mathcal{H}}\bigl({[\mathrm{GABA}]_{\mathrm{e}}},K_{\gamma}^{{\mathrm{ae}}} \bigr) -V_{\gamma}^{{\mathrm{ne}}} {\mathcal{H}}\bigl({[\mathrm{GABA}]_{\mathrm{e}}},K_{\gamma}^{{\mathrm{ne}}} \bigr), \end{aligned}$$

5i $$\begin{aligned} \frac{d {[\mathrm{GABA}]_{\mathrm{a}}}}{dt} =& V_{\gamma}^{{\mathrm{ae}}} {\mathcal {H}}\bigl({[ \mathrm{GABA}]_{\mathrm{e}}},K_{\gamma}^{{\mathrm{ae}}} \bigr)-V_{\gamma}^{c} {[\mathrm{GABA}]_{\mathrm{a}}}. \end{aligned}$$

In the sigmoidal functions for ${[\mathrm{Glu}]_{\mathrm{e}}}$ dynamics (5e), parameters $V_{G}^{{\mathrm{ae}}}$ and $V_{G}^{{\mathrm{ne}}}$ are the maximum rates of glutamate uptakes by the astrocytes and the neurons, respectively, $s_{g}$ represents the activation threshold and $r_{g}$ the sigmoidal slope at the inflection point. Parameters $V_{\gamma}^{{\mathrm{ae}}}$ and $K_{\gamma }^{{\mathrm{ae}}}$ (resp. $V_{\gamma}^{{\mathrm{ne}}}$ and $K_{\gamma}^{{\mathrm{ne}}}$) in Eq. (5h) are, respectively, the maximum rate and concentration for astrocyte (resp. neural) GABA transporter. Finally, $V_{G}^{c}$ and $V_{\gamma}^{c}$ are the glutamate and GABA degradation rates in astrocytes. We refer the reader to [30] for a detailed explanation of the dynamics.

System (5a)-(5i) is built as a feedforward coupling of the neural compartment onto the astrocyte one. Hence, in this model, the neural compartment is not impacted by the neurotransmitter concentrations in the extracellular space. As mentioned in the introduction, these concentrations have been proven to modulate the local neuron excitability and this feedback has been identified in recent studies [1] to be an essential mechanism of several pathologies triggered by astrocytic uptake deficiencies. Consequently, our aim is to include such feedback in the model in order to study the effects of different astrocyte dysfunctioning on the neuronal activity.

### Astrocytic Feedback and Neuron–Astrocyte Mass Model

The concentrations of neurotransmitters in a synaptic cleft act on the excitability threshold of the postsynaptic neuron. In the neuron–astrocyte model (5a)-(5i) the alteration of this neural excitability threshold can be reproduced by dynamical changes in $v_{P}$, $v_{P'}$ and $v_{I}$. In the following, we describe how we model the modulation of the neuron excitability in each population by the neurotransmitter concentrations in the extracellular space basing ourselves on biological knowledge.

Extracellular concentrations of neurotransmitters have a thresholded impact on neural activity [1]. Precisely, on one hand, the impact of neurotransmitter concentrations on neural activity is implicitly taken into account in the neuronal compartment, thus the astrocyte feedback steps in only when the concentrations become larger than physiological ones. On the other hand, the postsynaptic neurons are saturated when these concentrations become too large and, consequently, the neural excitability remains bounded. As explained in the introduction, quantitative experimental data of the impact of neurotransmitter concentrations on neural excitability do not exist up to now. For fixing ideas, we consider sigmoidal functions to model the astrocyte feedback on neural excitability which is a natural choice for aggregating the qualitative experimental knowledge. Yet the upcoming mathematical analysis can easily be extended to any bounded increasing functions with a unique inflection point.

We introduce three sigmoidal functions to model the components of the astrocyte feedback:

$$\begin{aligned} (\mathrm{a}) &\quad m_{G}^{P} {\mathcal{S}}\bigl({[ \mathrm{Glu}]_{\mathrm{e}}}, v_{G}, r_{G}\bigr) \quad \text{for glutamate feedback on pyramidal neurons}, \\ (\mathrm{b}) &\quad m_{G}^{I} {\mathcal{S}}\bigl({[ \mathrm{Glu}]_{\mathrm{e}}}, v_{G}, r_{G}\bigr) \quad \text{for glutamate feedback on interneurons}, \\ (\mathrm{c}) &\quad m_{\gamma} {\mathcal{S}}\bigl({[\mathrm{GABA}]_{\mathrm{e}}}, v_{\gamma }, r_{\gamma}\bigr) \quad \text{for GABA feedback on pyramidal neurons}. \end{aligned}$$

Note that glutamate binding mechanisms are the same for both pyramidal neurons and interneurons since both cell types express the same type of transporters [10, 37]. Thus, only parameters $m_{G}^{P}$ and $m_{G}^{I}$ representing the maximum coupling gains of the glutamate-dependent component of the astrocyte feedback discriminate between the coupling functions $m_{G}^{P} {\mathcal{S}}({[\mathrm{Glu}]_{\mathrm{e}}}, v_{G}, r_{G})$ and $m_{G}^{I} {\mathcal{S}}({[\mathrm{Glu}]_{\mathrm{e}}}, v_{G}, r_{G})$, insofar as the number of transporters is cell-specific and so is the ensuing uptake rate. Model parameters are summarized in Table 1 and their values used in the simulations of this study were chosen to qualitatively reproduce typical physiological data.

**Table 1:** **Descriptions and values of the neuron–astrocyte mass model parameters**

Parameter	Interpretation	Value
*Neuronal compartment*		
*A*	Average excitatory synaptic gain	$3.25~\mbox{mV}$
*B*	Average inhibitory synaptic gain	$22~\mbox{mV}$
$\frac{1}{a}$	Time constant of excitatory postsynaptic potentials	$\frac{1}{100}~\mbox{s}$
$\frac{1}{b}$	Time constant of inhibitory postsynaptic potentials	$\frac{1}{50}~\mbox{s}$
$e_{0}$	Half of the maximum discharge rate of a neuronal population	$2.5~\mbox{s}^{-1}$
$v_{0}$	Basic excitability threshold for neurons	$6~\mbox{mV}$
*r*	Stiffness of neuronal excitability	$0.56~\mbox{mV}^{-1}$
$C_{1}$	Strength of the synaptic connections from *P* to $P'$	135
$C_{2}$	Strength of the synaptic connections from $P'$ to *P*	108
$C_{3}$	Strength of the synaptic connections from *P* to *I*	33.75
$C_{4}$	Strength of the synaptic connections from *I* to *P*	33.75
*G*	Gain of the direct excitatory feedback from *P* to itself	40
*Glial compartment*		
*W*	Tunes the peak amplitude of glutamate concentrations	$53.6~\mu\mbox{M}\cdot\mbox{s}^{-1}$
*Z*	Tunes the peak amplitude of GABA concentrations	$53.6~\mu\mbox{M}\cdot\mbox{s}^{-1}$
$w_{1}$	Tune the rise and decay times of glutamate release transfer function	$90~\mbox{s}^{-1}$
$w_{2}$		$33~\mbox{s}^{-1}$
$z_{1}$	Tune the rise and decay times of GABA release transfer function	$90~\mbox{s}^{-1}$
$z_{2}$		$33~\mbox{s}^{-1}$
$V_{G}^{{\mathrm{ne}}}$	Maximal rate of glutamate uptake by neurons	$0.5~\mu\mbox{M}\cdot\mbox{s}^{-1}$
$V_{G}^{{\mathrm{ae}}}$	Maximal rate of glutamate uptake by astrocytes	$4.5~\mu\mbox{M}\cdot\mbox{s}^{-1}$
$s_{g}$	Activation threshold of sigmoid glutamate uptakes	$6~\mu\mbox{M}$
$r_{g}$	Stiffness of sigmoid glutamate uptakes	$0.9~\mu\mbox{M}^{-1}$
$V_{\gamma}^{{\mathrm{ae}}}$	Maximal rate of astrocyte GABA uptake	$2~\mu\mbox{M}\cdot\mbox{s}^{-1}$
$K_{\gamma}^{{\mathrm{ae}}}$	Maximal concentration for Hill dynamics of astrocyte GABA uptake	$8~\mu\mbox{M}$
$V_{\gamma}^{{\mathrm{ne}}}$	Maximal rate of neuronal GABA uptake	$5~\mu\mbox{M}\cdot\mbox{s}^{-1}$
$K_{\gamma}^{{\mathrm{ne}}}$	Maximal concentration for Hill dynamics of neuronal GABA uptake	$24~\mu\mbox{M}$
$V_{G}^{c}$	Rate of glutamate degradation by astrocytes	$9~\mu\mbox{M}\cdot\mbox{s}^{-1}$
$V_{\gamma}^{c}$	Rate of GABA degradation by astrocytes	$9~\mu\mbox{M}\cdot\mbox{s}^{-1}$
*Neuron excitability modulations by neurotransmitter concentrations* (*feedbacks*)		
$v_{G}$	Excitability threshold of glutamate feedback function	$30~\mu\mbox{M}$
$r_{G}$	Stiffness of sigmoid glutamate feedback function induced by glutamate	$0.15~\mu\mbox{M}^{-1}$
$m_{G}^{P}$	Maximal coupling gain of glutamate feedback on pyramidal neurons	$2.5~\mbox{mV}$
$m_{G}^{I}$	Maximal coupling gain of glutamate feedback on interneurons	$1~\mbox{mV}$
$v_{\gamma}$	Excitability threshold of GABA feedback function	$25~\mu\mbox{M}^{-1}$
$r_{\gamma}$	Stiffness of sigmoid GABA feedback function	$0.12~\mu\mbox{M}^{-1}$
$m_{\gamma}$	Maximal coupling gain of GABA feedback	$1~\mbox{mV}$

We previously mentioned that astrocyte feedback acts on the excitability thresholds of neurons. More specifically, if there is an excess of neurotransmitter in a synapse from a neuron ${{\mathrm{n}}}_{1}$ of population ${{\mathrm{N}}}_{1}$ to a neuron ${{\mathrm{n}}}_{2}$ of population ${{\mathrm{N}}}_{2}$, the extracellular concentration of neurotransmitter acts on the postsynaptic neuron ${{\mathrm{n}}}_{2}$ by changing its excitability threshold. In system (5a)-(5i) the excitability threshold of neurons, that is, a parameter at the single cell scale, does not appear explicitly. However, when the excitability of the population ${{\mathrm{N}}}_{2}$ neurons changes at the individual scale, the number of neurons activated in this population by a given input changes as well and so does the output of this population. Following the mass approach, we scale this feature to the population, and, consequently, we choose to change parameter $v_{{{\mathrm{N}}}_{2}}$ in the equation corresponding to the output of population ${{\mathrm{N}}}_{2}$ since this parameter represents a modulation of the threshold of the sigmoidal function $\hat{\mathcal{S}}$.

Let us now describe how we build the feedbacks on the dynamics of the neural compartment using the sigmoidal functions of the neurotransmitter concentrations. The dashed arrows in Fig. 3 illustrate these feedbacks. We need to consider separately each type of synapse in the NMM, and the variables $x_{0}$, $x_{1}$, and $x_{2}$ for the feedbacks building. The NMM embeds five types of synaptic connections between populations: (i) $P' \to P$, (ii) $P \to P'$, (iii) $P \to I$, (iv) $I \to P$, (v) $P \to P$. In the following we detail the modulation of neural intermediary variables for each kind of synapse separately, then we gather these changes to specify the coupling terms reproducing the astrocyte feedback.

In the framework of the local neuron–astrocyte mass model, the astrocyte feedback does not impact the synaptic connections of type $P' \to P$ or $P \to P'$. As a matter of fact, the astrocyte compartment only takes into account neurotransmitters released locally by neurons of populations *P* and *I*, whereas population $P'$ is nonlocal to population *P*. Hence, extracellular concentrations of neurotransmitters in the vicinity of $P'$ have no impact on the neuronal activity of *P* and the concentrations in the neighborhood of *P* and *I* do not influence the postsynaptic neurons of population $P'$.

A synaptic connection of type $P \to I$ concerns the variable $x_{2}$. As shown in [1, 38], in the case of glutamate excess in the extracellular space, the postsynaptic neuron is more excitable. Consequently, more neurons are activated in the population *I*. We model this mechanism at the mesoscopic scale by introducing a dependency of population *I* excitability threshold $v_{I}$ on the extracellular glutamate concentration and set in Eq. (2c):

$$v_{I} = v_{0}-m_{G}^{I} { \mathcal{S}}\bigl({[\mathrm{Glu}]_{\mathrm{e}}}, v_{G}, r_{G}\bigr). $$

While experiments have evidenced impacts of extracellular glutamate concentrations on the excitability of GABAergic neurons [38], we lack experimental data and conclusions on the way GABA acts on the excitability of each subtypes of GABAergic neurons. In particular, observations raise the question of whether all subtypes are subject to mechanisms of GABA-mediated excitability modulation in the same way [39, 40]. Since our model is not subtype-specific for the sake of compactness, we chose not to embed any GABA feedback on interneuron population dynamics.

On the one hand, a synaptic connection of type $I \to P$ is concerned by extracellular concentrations of GABA since it involves GABAergic interneurons. In case of a GABA excess in the extracellular space, the inhibition of the postsynaptic neuron is strengthened (see e.g. [1]), *i.e.* less neurons are activated in population *P*, which is translated in the NMM by an increase of the threshold of the sigmoidal term in the $x_{0}$ dynamics. On the other hand, a synaptic connection of type $P \to P$ is impacted by the extracellular concentration of glutamate implying a modulation of variable $x_{0}$ dynamics as well. In the case of an excess of glutamate in this kind of synapse, the postsynaptic neuron is more excitable [1]. Hence, more neurons are activated in population *P* which can be reproduced by a decrease in the threshold parameter appearing in (3d). Gathering both modulations impacting the excitability of population *P*, we set in Eq. (3d)

$$v_{P} = v_{0}+m_{\gamma} {\mathcal{S}}\bigl({[ \mathrm{GABA}]_{\mathrm{e}}}, v_{\gamma}, r_{\gamma} \bigr)-m_{G}^{P} {\mathcal{S}}\bigl({[\mathrm{Glu}]_{\mathrm{e}}}, v_{G}, r_{G}\bigr). $$

The new neuron–astrocyte mass model embedding the astrocyte feedback is obtained from model (5a)-(5i) by considering the dynamical entries $v_{I}$ and $v_{P}$ mentioned above. Accordingly, the sigmoidal functions appearing in Eqs. (5a), (5b), (5c), (5d), and (5g) become

$$\begin{aligned} \hat{\mathcal{S}}(y_{1}-y_{2},v_{P}) =& \hat{ \mathcal {S}}\bigl(y_{1}-y_{2},v_{0}+m_{\gamma} {\mathcal{S}}\bigl({[\mathrm{GABA}]_{\mathrm{e}}}, v_{\gamma }, r_{\gamma}\bigr) \\ &{}-m_{G}^{P} {\mathcal{S}}\bigl({[ \mathrm{Glu}]_{\mathrm{e}}}, v_{G}, r_{G}\bigr)\bigr), \\ \hat{\mathcal{S}}(C_{1} y_{0},v_{P'}) =& \hat{ \mathcal{S}}(C_{1} y_{0},v_{0}), \\ \hat{\mathcal{S}}(C_{3} y_{0},v_{I}) =& \hat{ \mathcal{S}}\bigl(C_{3} y_{0},v_{0}-m_{G}^{I} {\mathcal{S}}\bigl({[\mathrm{Glu}]_{\mathrm{e}}}, v_{G}, r_{G}\bigr)\bigr). \end{aligned}$$

### Bifurcation-Based Characterization of the Neural Activity: The Case of Noise-Induced Spiking

The behavior of NMMs can be deduced from the bifurcation diagram according to the value $p(t)=p$ considered as a parameter, as it has been performed in [28] on the Jansen–Rit model. In [29], we have classified the types of time-series patterns generated by model (3a)-(3f) and the associated bifurcation structures according to the strengths of the different excitatory inputs to population *P*. Let us recall the bifurcation diagram underlying the predominant type of generated time series, which we will consider in this article.

**Fig. 4:**
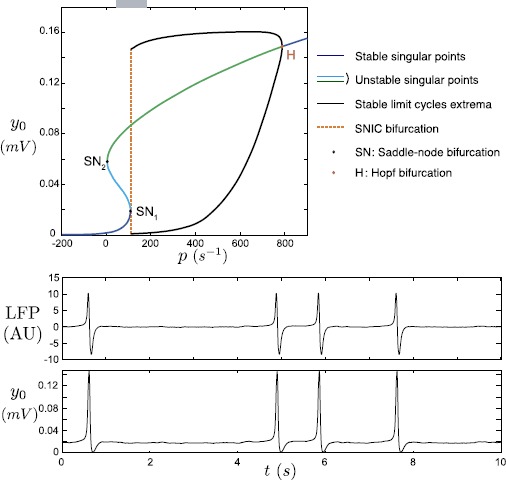
Bifurcation diagram according to *p* (*left*) and associated LFP and $y_{0}$ time series (*right*). *Blue curves*: stable singular points. *Cyan* (resp. *green*) *curves*: singular points with one (resp. two) eigenvalues with positive real parts. *Black curves*: $y_{0}$ extrema along stable limit cycles. *Black points* ($\mathrm{SN}_{1}$ and $\mathrm{SN}_{2}$): saddle-node bifurcations. *Red point* ($\mathrm{H}_{1}$): supercritical Hopf bifurcation. *Dashed orange line*: Saddle-Node on Invariant Circle (SNIC) bifurcation. *Horizontal gray bar*: confidence interval of the Gaussian variable $p(t)$ used to generate the time series i.e. $[\bar{p}-\sigma, \bar{p}+\sigma]$ where *p̄* and *σ* are the mean and the variance of the associated normal distribution, respectively

Model (3a)-(3f) has the following useful features [29]. First, for a fixed value of parameter *p*, the $y_{0}$ value of a singular point suffices to have explicit expressions of all the other components. Second, for a given $y_{0}$ value, there exists a unique value of *p* such that $y_{0}$ corresponds to a singular point. In other terms, the set of singular points obtained for the different values of *p* is a graph over $y_{0}$. Hence, we can visualize the shape of the singular point locus in the plane $(p,y_{0})$: in the case presented here (see top panel of Fig. 4), this curve of singular points is S-shaped. In the following description, for a given bifurcation “*X*” according to parameter *p*, we denote by $p_{X}$ the bifurcation value and, if the bifurcation involves a singular point, we denote by $y_{X}$ the corresponding $y_{0}$ value.

Two saddle-node bifurcations $\mathrm{SN}_{1}$ and $\mathrm{SN}_{2}$ split the curve of singular points into three branches. We name “lower branch”, “middle branch”, and “upper branch” the sets of singular points, respectively, satisfying $y_{0}< y_{{\mathrm{SN}}_{1}}$, $y_{{\mathrm{SN}}_{1}}< y_{0}< y_{{\mathrm{SN}}_{2}}$, and $y_{0}>y_{{\mathrm{SN}}_{2}}$. Singular points on the lower branch are stable (blue) and those on the middle branch are unstable (cyan). Singular points on the upper branch are unstable (green) for $p< p_{{\mathrm{H}}_{1}}$ and stable (blue) otherwise. At $p=p_{{\mathrm{H}}_{1}}$ the system undergoes a supercritical Hopf bifurcation $\mathrm{H}_{1}$ giving birth to a stable limit cycle for $p< p_{{\mathrm{H}}_{1}}$ that persists until $p=p_{{\mathrm{SN}}_{1}}=p_{{\mathrm{SNIC}}}$ where it disappears by a saddle-node on invariant circle (SNIC) bifurcation (dashed orange line). The existence of the SNIC bifurcation is essential because it implies the appearance of a large amplitude stable limit cycle with large period. Hence, according to the value of *p*, the system alternates between oscillatory phases (for $p>p_{{\mathrm{SNIC}}}$) and quiescent phases (for $p< p_{{\mathrm{SNIC}}}$). In other terms, $p_{{\mathrm{SNIC}}}$ plays the role of an activation threshold for the neural compartment.

This means that, given a constant input $p(t)=\bar{p}$ such that $\bar {p} < p_{{\mathrm{SNIC}}}$, we expect the NMM to reproduce quiescent neuronal activity. On the other hand, if we consider Gaussian noise as input that is normally distributed with *p̄* as mean value and large enough variance *σ*, the NMM output results instead in an alternation of quiescent phases and isolated LFP spikes (see Fig. 4), consistent with emergence of Noise-Induced Spiking (NIS) [29]. Importantly, NIS frequency depends on the input noise statistics (*i.e.* mean and variance in the case of Gaussian noise) as reflected by the confidence interval $[\bar{p}-\sigma, \bar {p}+\sigma]$ reported by the gray bar above the bifurcation diagram on the left panel of Fig. 4.

Such LFP activity, *i.e.* sparse large amplitude spikes, corresponds to episodic synchronization of the neuron activities among the populations. This pattern of activity is symptomatic of a strong excitability of the neuronal system that can turn into hyperexcitability during pathological crisis. In [29] we have fitted the NMM parameter values for reproducing interictal and pre-ictal spiking activities by tuning the coupling gain of the indirect excitatory feedback. We have compared these outputs to experimental data recorded from epileptic mice (Mesial Temporal Lobe Epilepsy mouse model). It appeared that the NIS behavior is well suited for reproducing the high amplitude sharp waves characterizing the activity pattern recorded between pathological crises. For these fixed parameter values of the NMM, the activity is stable in the sense that the oscillation frequency does not change much with time. The values of the parameters associated with the NMM used in this study and given in Table 1 have been chosen so as to reproduce NIS behavior using the analysis in [29]. In the following, we study the variations of the activity when the neural dynamics is altered by the surrounding activity, *i.e.* the astrocyte feedback.

### NMM Dynamics: Example from GABA Injection

To illustrate the impact of the astrocytic feedback on neural excitability and introduce the dynamical tools that we are using in the remainder of the article, we set to study LFP dynamics generated by our model both with and without astrocytic feedback, leveraging on the role of the SNIC bifurcation occurring at $p=p_{{\mathrm{SNIC}}}$ on the emergence of different regimes of neuronal firing.

**Fig. 5:**
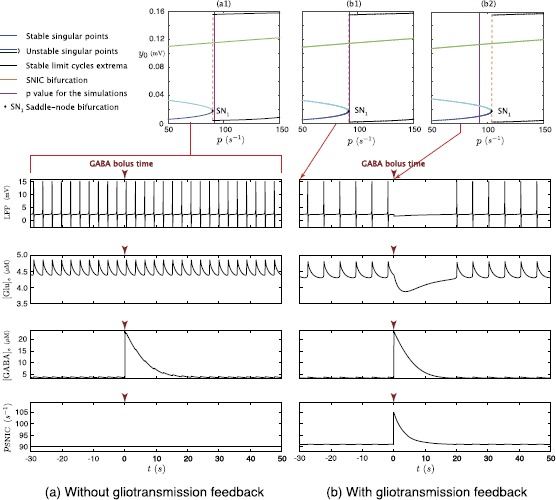
Comparison of the effects of a GABA bolus on the feedforward and bilaterally coupled neuron–astrocyte mass models. Bifurcation diagrams according to *p* (*top panels*) computed for the model without feedback for all *t* (**a1**) and for the model with feedback at $t=0~\mbox{s}$ (**b1**) and $t=30~\mbox{s}$ (**b2**). Time series (*bottom panels*) corresponding to LFP, glutamate and GABA extracellular concentrations and time variations of $p_{{\mathrm{SNIC}}}$ generated by the models without feedback (*left*) and with feedback (*right*). *The purple lines* on the bifurcation diagrams materialize the fixed value of input *p*. *The dark red arrows* above each time series materialize the time of GABA bolus injection

With this aim, we simulate injection of GABA ($20~\mbox{AU}$) into the extracellular space at $t=0$. Assuming $p(t)=p \gtrapprox p_{{\mathrm{SNIC}}}$ (Fig. 5, panels (a1) and (b1)). Either in the presence of astrocytic feedback or lack thereof, the model generates low-frequency NIS right after injection time. Nonetheless it may be noted that, in the presence of astrocytic feedback, even if extracellular GABA concentration is low—and the corresponding sigmoidal feedback is consequently very weak—this is sufficient to affect neural activity—as reflected by the difference in spike frequency between the two LFP time series.

In the time series generated by the model without feedback (left panels of Fig. 5), the neural activity and the glutamate concentration dynamics remain unchanged (Fig. 5(a)) after the instantaneous increase of ${[\mathrm{GABA}]_{\mathrm{e}}}$ at $t=0$, which mimics GABA injection. In contrast, in the model with astrocyte feedback (right panels of Fig. 5), the strong increase of $[\mathrm{GABA}]_{\mathrm{e}}$ halts neuronal activity, which, in turn reduces synaptic release of glutamate. Once $[\mathrm{GABA}]_{\mathrm{e}}$ has become sufficiently low, neural activity re-emerges and both glutamate and GABA concentrations come back to their respective basal values, oscillating with neural activity.

These phenomena can be well accounted by arguments based on bifurcation analysis (top panels of Fig. 5). In the model without astrocytic feedback, neural dynamics is decoupled from astrocytic dynamics. Hence, the bifurcation diagram of the NMM remains unchanged during all simulated time (Fig. 5(a1)). In this fashion, the excitability threshold $p_{{\mathrm{SNIC}}}$ of the neural compartment is constant in time (Fig. 5, bottom panel). On the other hand, in the system with feedback, $v_{P}$, $v_{P'}$ and $v_{I}$ depend on GABA and glutamate concentrations. Consequently, the bifurcation diagram for the neural compartment is time dependent, and so is the value of $p_{{\mathrm{SNIC}}}$, which changes along with the astrocytic variables. Thus, right after GABA injection, $p_{{\mathrm{SNIC}}}$ is larger than the input value *p* (Fig. 5(b2)), and because $p_{{\mathrm{SNIC}}}$ plays the role of activation threshold for the neural population, then as long as GABA concentration remains high, the neural population remains quiescent. One may easily see by a simple algebraic calculation (not shown) that the astrocytic compartment admits a unique singular stable point for any neural state. Hence, starting from a high extracellular GABA concentration, this concentration decreases toward the attractive state slowly decreasing $p_{{\mathrm{SNIC}}}$, as shown in the bottom panel of Fig. 5, case (b). Once, GABA is low enough, $p_{{\mathrm{SNIC}}}$ becomes smaller than *p*, and the system oscillates again.

This analysis shows how the model with feedback can take into account changes in glutamate and GABA dynamics to modify all the dynamics of the system and illustrates the interest of embedding the astrocyte feedback in such neuron–astrocyte model. Our model allows us to study the effects of variations in glutamate or GABA dynamics on neural activity. In the following, we study the effects of deficiencies in the uptake of neurotransmitters by the astrocytes both on extracellular concentrations and neural activity. For both types of deficiency (GABA and glutamate uptake), we first describe the biologic context and mechanisms and their outcomes, then we provide a mathematical analysis of the underlying dynamical mechanisms to explain the effects that can be expected in a biological system.

## Deficiency of Astrocytic GABA Uptake

We now set to investigate some predictions of our model, starting from the consideration of the scenario of deficiency of astrocytic GABA transporters whose experimental correlate is a low capacity by astrocytes to uptake extracellular GABA [7]. Because a single astrocyte may ensheath hundreds of thousands of synapses [41, 42], the increase of extracellular GABA resulting from slow, defective astrocytic uptake is expected to promote neuronal inhibition and thus reduces synaptic release in line with what is elucidated in the previous Sect. 2.5. Thus, once we consider many defective astrocytes, all neurons in the proximity of these astrocytes are likely to be inhibited [43].

To simulate deficiency of astrocytic GABA uptake in our model, we decrease the maximum (saturated) rate of astrocytic GABA uptake from the extracellular space ($V_{\gamma}^{{\mathrm{ae}}}$) as this parameter directly depends on the uptake efficiency of astrocytic GABA transporters. At the neuronal population level we are interested instead in deriving the dependence of $p_{{\mathrm{SNIC}}}$ on astrocytic feedback. With this aim, because glutamate binding by neuronal receptors is independent of cell types, we may set:

$$\begin{aligned} m_{G}^{I} {\mathcal{S}}\bigl({[\mathrm{Glu}]_{\mathrm{e}}}, v_{G}, r_{G}\bigr) \equiv& v_{1}, \\ m_{G}^{P} {\mathcal{S}}\bigl({[\mathrm{Glu}]_{\mathrm{e}}}, v_{G}, r_{G}\bigr) \equiv& \frac {m_{G}^{P}}{m_{G}^{I}} v_{1}, \\ m_{\gamma} {\mathcal{S}}\bigl({[\mathrm{GABA}]_{\mathrm{e}}}, v_{\gamma}, r_{\gamma}\bigr) \equiv& v_{2}, \end{aligned}$$

where $v_{1}$ and $v_{2}$ are defined within the boundaries of $m_{G}^{I} {\mathcal{S}}({[\mathrm{Glu}]_{\mathrm{e}}}, v_{G}, r_{G})$ and $m_{\gamma} {\mathcal {S}}({[\mathrm{GABA}]_{\mathrm{e}}}, v_{\gamma}, r_{\gamma})$, that is, $0 \leq v_{1} \leq m_{G}^{I}$ and $0 \leq v_{2} \leq m_{\gamma}$.

With these new notations, the dynamical excitability thresholds $v_{P}$, $v_{P'}$, and $v_{I}$ of populations *P*, $P'$, and *I* become

$$\begin{aligned} v_{P} =& v_{0}+v_{2}-\frac{m_{G}^{P}}{m_{G}^{I}} v_{1}, \\ v_{P'} =& v_{0}, \\ v_{I} =& v_{0}-v_{1}. \end{aligned}$$

**Fig. 6:**
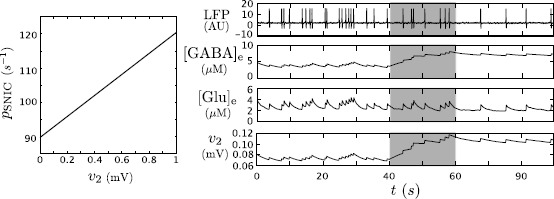
The dependency of the SNIC value on $v_{2}$ explains the impact of an alteration of the GABA astrocytic uptake on the neural activity. Variation of $p_{{\mathrm{SNIC}}}$ value according to $v_{2}$ (*left*). Time series corresponding to LFP, ${[\mathrm{GABA}]_{\mathrm{e}}}$, ${[\mathrm{Glu}]_{\mathrm{e}}}$ and $v_{2}=m_{\gamma} {\mathcal{S}}({[\mathrm{GABA}]_{\mathrm{e}}}, v_{\gamma }, r_{\gamma})$ (*right from top to bottom*) for $p(t)$ a Gaussian variable. At $t=40~\mbox{s}$, the GABA astrocytic uptake is artificially altered by setting $V_{\gamma}^{{\mathrm{ae}}}=0$ for any subsequent time. *The gray time window* highlights the transient toward the new behavior

By these new parameters, an increase (decrease) of extracellular GABA or glutamate are represented by an increase (decrease) of $v_{2}$ or $v_{1}$, respectively. Accordingly, because a deficiency of astrocytic GABA uptake increases extracellular GABA concentration, then we aim at characterizing the dependency of $p_{{\mathrm{SNIC}}}$ on the value of $v_{2}$. In particular we formalize our first model prediction in Proposition 3.1 and exemplify it by numerical continuation of the SNIC bifurcation for varying $v_{2}$ in Fig. 6 (left panel). In carrying out our following analysis, we assume $v_{1}$ constant, leaving justification of such assumption *a posteriori* at the end of the section.

### Proposition 3.1

*The threshold for neuronal activation*, $p_{{\mathrm{SNIC}}}$, *linearly increases with the GABA*-*induced modulation of neural excitability* $v_{2}$.

### Proof

The set of the system singular points obtained for the different values of parameter *p* can be explicitly expressed according to $y_{0}$, $v_{1}$, and $v_{2}$ all other parameters being fixed. Using system (3a)-(3f), a singular point satisfies $y_{3}=y_{4}=y_{5}=0$. Under these conditions, annihilation of the r.h.s. of Eq. (3f) allows expressing $y_{2}$ with respect to $y_{0}$. Then annihilation of the r.h.s. of (3d) provides $y_{1}$ as function of $y_{0}$. Finally, by annihilation of Eq. (3e), we find that the $y_{0}$ components of the singular points for given values of *p*, $v_{1}$ and $v_{2}$ are characterized as solutions of

6 $$ p = f(y_{0},v_{1},v_{2}), $$

where

7 $$\begin{aligned} f(y_{0},v_{1},v_{2}) =& \frac{a}{A} \biggl(v_{0}-\frac{m_{G}^{P}}{m_{G}^{I}} v_{1}+v_{2} \biggr)-\frac{a}{A r}{{\mathrm{ln}}} \biggl( \frac{2 A e_{0}-a y_{0}}{a y_{0}} \biggr) - \frac{a G}{A} y_{0} \\ &{} -C_{2} \hat{\mathcal{S}}(C_{1} y_{0},v_{0}) +\frac{a B}{b A} C_{4} \hat {\mathcal{S}}(C_{3} y_{0},v_{0}-v_{1}). \end{aligned}$$

All the other components of a given singular point can be explicitly expressed as functions of the $y_{0}$ component. The calculation is straightforward from the annihilation of the vector field (5a)-(5i) as explained above. Since these components are not needed for the following analysis and are only minor calculus details, we do not specify these heavy expressions. We rewrite Eq. (7) as follows:

8 $$ f(y_{0},v_{1},v_{2})= \frac{a}{A} v_{2}+q(y_{0},v_{1}). $$

Obviously the two saddle-node bifurcation values $p_{{\mathrm{SN}}_{1}}$ and $p_{{\mathrm{SN}}_{2}}$ are relative extrema of $f(y_{0},v_{1},v_{2})$. In particular $p_{{\mathrm{SN}}_{1}}=p_{{\mathrm{SNIC}}}$ coincide with the local maximum of $f(y_{0},v_{1},v_{2})$ and, as such, is of the solution of

9a $$\begin{gathered} p = f(y_{0},v_{1},v_{2}), \end{gathered}$$

9b $$\begin{gathered} \frac{\partial f}{\partial y_{0}}(y_{0},v_{1},v_{2}) = 0, \end{gathered}$$

9c $$\begin{gathered} \frac{\partial^{2} f}{\partial y_{0}^{2}}(y_{0},v_{1},v_{2}) \leqslant0. \end{gathered}$$

Since $\frac{\partial f}{\partial y_{0}}(y_{0},v_{1},v_{2})$ is independent on $v_{2}$, so is $y_{{\mathrm{SNIC}}}$ and it can be considered as a parameter in Eq. (9a). From (8) and (9a)-(9c) we obtain the following expression for $p_{{\mathrm{SNIC}}}$:

$$p_{{\mathrm{SNIC}}} = \frac{a}{A} v_{2}+q(y_{{\mathrm{SNIC}}},v_{1}). $$

 □

Let us consider the model generating an oscillatory output with a fixed value of *p* ($p_{{\mathrm{SNIC}}}< p$). If the extracellular concentration of GABA increases (e.g. by an injection of GABA as in Fig. 5), the value of $v_{2}$ increases and Proposition 3.1 asserts that the value of $p_{{\mathrm{SNIC}}}$ also increases. As already explained, the closest $p_{{\mathrm{SNIC}}}$ is to *p*, with $p_{{\mathrm{SNIC}}}< p$, the largest is the limit cycle period, thus the oscillation frequency of the outputs decreases. If $p_{{\mathrm{SNIC}}}$ increases enough such that $p_{{\mathrm{SNIC}}}>p$, the stable limit cycle of the system disappears, and the neural compartment becomes quiescent.

In the case of deficiency of GABA uptake, the extracellular concentration of GABA increases, and we can use Proposition 3.1 to explain the following effects. For that purpose, we use the following *in silico* protocol: we initialize the neuron–astrocyte model in an oscillatory phase with a low oscillation frequency and consider $p(t)$ a Gaussian input. At $t=40~\mbox{s}$, we turn off the GABA astrocyte uptake by setting $V_{\gamma}^{{\mathrm{ae}}}=0$ (Fig. 6). The result is an increase in GABA extracellular concentration implying an increase in $p_{{\mathrm{SNIC}}}$. As $p_{{\mathrm{SNIC}}}$ increases, the probability for $p(t)$ to overcome $p_{{\mathrm{SNIC}}}$ along the associated Brownian motion decreases, and also does the oscillation frequency (Fig. 6). Consequently, we observe a decrease in the oscillation frequency after $t>40~\mbox{s}$. In the time series, the oscillation frequency decreases gradually during a transient ($40~\mbox{s}< t<60~\mbox{s}$) until reaching its minimum. This can be explained by the slow increase of GABA extracellular concentration that reaches its new baseline at $t=60~\mbox{s}$.

To summarize, our hitherto analysis reveals that a deficiency of astrocytic GABA uptake causes a decrease of neural firing, leaving extracellular glutamate concentration close to its baseline. This emphasizes that, under such conditions, changes in glutamate-induced modulation of neuronal excitability ($v_{1}$) are overall negligible insofar as neural activity is sufficiently inhibited by high extracellular GABA.

## Deficiency of Astrocytic Glutamate Uptake

We now focus on the impact of deficient astrocytic glutamate uptake on neuronal activation. This scenario results in an increase of extracellular glutamate which in principle is expected to make neurons more excitable and thus more prone to fire. However, such increase in excitability may be (at least partly) counteracted by the resulting boost in interneurons firing which triggers synaptic release of GABA, overall increasing extracellular levels of this species and promoting, in turn, neural inhibition. This suggests that the possible balance between glutamatergic neuronal excitation and GABAergic inhibition may lead to nontrivial dynamics regimes for neural activity. Considering the rationale outlined in the previous section, we now set out to characterize the changes in the SNIC bifurcation value $p_{{\mathrm{SNIC}}}$ (*i.e.* the threshold for neural activation) with $v_{1}$ value. The latter variable, introduced in the beginning of Sect. 3, quantifies the instantaneous impacts of glutamate-induced modulations on both interneuron and pyramidal neuron excitabilities. Hence, it depends directly on the astrocytic glutamate uptake. We therefore analyze the various consequence on the neural activity of a deficiency of astrocytic glutamate uptake simulated by choosing small values for the maximum uptake rate $V_{G}^{\mathrm{ae}}$.

We note in this regard, however, that, for certain parameter sets of the whole model, the SNIC bifurcation may be lost as $v_{1}$ changes. Due to the high dimensionality of our parameter space, it is difficult to estimate the region of existence of the SNIC for any value of $v_{1}$, yet by the arguments exposed in [29] we may safely state that this region is large enough to be always found for our choice of parameters (see Table 1). Accordingly, in the following, we assume that $p_{{\mathrm{SNIC}}}$ always exists and the associated saddle-node bifurcation is not degenerate for any $v_{1}$ such as $0 \leq v_{1} \leq m_{G}^{I}$, that is the maximum interval of values taken by $v_{1} = m_{G}^{I} {\mathcal{S}}({[\mathrm{Glu}]_{\mathrm{e}}}, v_{G}, r_{G})$.

We recall that $p_{{\mathrm{SNIC}}}$ can be written as follows:

10 $$ p_{{\mathrm{SNIC}}} = f(y_{{\mathrm{SNIC}}},v_{1},v_{2}), $$

where *f* is given by (7). Since we consider $v_{2}$ fixed we introduce the function

$$g(y_{0},v_{1}) \equiv f(y_{0},v_{1},v_{2})|_{v_{2} = \mathrm{const}}. $$

As explained above, for each $v_{1}$, there exists a unique bifurcation value $p_{{\mathrm{SNIC}}}$ occurring at the non-hyperbolic (saddle-node) singular point characterized by $y_{{\mathrm{SNIC}}}$, which is defined by

$$\begin{aligned} \frac{\partial g}{\partial y_{0}}(y_{{\mathrm{SNIC}}},v_{1}) =& 0 , \\ \frac{\partial^{2} g}{\partial y_{0}^{2}}(y_{{\mathrm{SNIC}}},v_{1}) < & 0. \end{aligned}$$

This value satisfies $p_{{\mathrm{SNIC}}} = g(y_{{\mathrm{SNIC}}},v_{1})$. We cannot find the explicit expressions of $y_{{\mathrm{SNIC}}}(v_{1})$ and $p_{{\mathrm{SNIC}}}(v_{1})$. Thus, for characterizing the variations of $p_{{\mathrm{SNIC}}}$ with $v_{1}$, we take advantage of the implicit definitions above and focus on localizing the extrema of $p_{{\mathrm{SNIC}}}(v_{1})$.

### Proposition 4.1

*Assume that*, *for all* $v_{1}$ *such that* $0 \leq v_{1} \leq m_{G}^{I}$, $p_{{\mathrm{SNIC}}}$ *exists and the associated saddle*-*node bifurcation is not degenerate*. *Note* $\chi= \frac{B e_{0} r C_{4}}{2 b}$. *Then*:

1. *if* $\frac{m_{G}^{P}}{m_{G}^{I}} \geqslant\chi$, $p_{{\mathrm{SNIC}}}(v_{1})$ *has no local extremum*,
2. *if* $0 < \frac{m_{G}^{P}}{m_{G}^{I}} < \chi$, $p_{{\mathrm{SNIC}}}(v_{1})$ *may admit two local extrema*: *a minimum at* $v_{1}^{*}$ *and a maximum at* $v_{1}^{**}$. *If both exist*, *then* $v_{1}^{*} < v_{1}^{**}$.

### Proof

Let us search for local extrema of the function $p_{{\mathrm{SNIC}}}(v_{1})$, which is implicitly defined by (9a)-(9c). Hence, we are interested in the following constrained optimization problems:

11 $$ \max \biggl\{ g(y_{0},v_{1}) \Bigm| \frac{\partial g}{\partial y_{0}}(y_{0},v_{1}) = 0 \biggr\} \qquad \biggl( \min \biggl\{ g(y_{0},v_{1}) \Bigm| \frac{\partial g}{\partial y_{0}}(y_{0},v_{1}) = 0 \biggr\} \biggr). $$

We introduce the associated Lagrangian function

$$\mathsf{L}(y_{0},v_{1},\lambda) = g(y_{0},v_{1})- \lambda \frac{\partial g}{\partial y_{0}}(y_{0},v_{1}). $$

The necessary condition for the existence of an extremum of *g* under the constraint $\frac{\partial g}{\partial y_{0}}=0$ is

$$\overrightarrow{\nabla}\mathsf{L}(y_{0},v_{1},\lambda)=0, $$

that is,

12a $$\begin{aligned} \frac{\partial g}{\partial y_{0}}(y_{0},v_{1})-\lambda \frac{\partial^{2} g}{\partial y_{0}^{2}}(y_{0},v_{1}) =&0, \end{aligned}$$

12b $$\begin{aligned} \frac{\partial g}{\partial v_{1}}(y_{0},v_{1})-\lambda \frac{\partial^{2} g}{\partial v_{1} \, \partial y_{0}}(y_{0},v_{1}) =&0, \end{aligned}$$

12c $$\begin{aligned} \frac{\partial g}{\partial y_{0}}(y_{0},v_{1}) =& 0. \end{aligned}$$

By assumption, the saddle-node bifurcation associated with the SNIC bifurcation is not degenerate, *i.e.* every solution of (11) such as $0 \leq v_{1} \leq m_{G}^{I}$ satisfies $\frac{\partial ^{2} g}{\partial y_{0}^{2}}(y_{0}, v_{1}) \neq0$. Thus, system (12a)-(12c) reads

13a $$\begin{aligned} \lambda =& 0, \end{aligned}$$

13b $$\begin{aligned} \frac{\partial g}{\partial v_{1}}(y_{0},v_{1}) =& 0, \end{aligned}$$

13c $$\begin{aligned} \frac{\partial g}{\partial y_{0}}(y_{0},v_{1}) =& 0. \end{aligned}$$

Therefore, if the problem under constraint admits an extremum, at this extremum it is $\frac{\partial g}{\partial v_{1}}=0$. Following the assumption that a SNIC bifurcation occurs for any value of $v_{1}$ between 0 and $m_{G}^{I}$, then Eq. (13c) admits a solution for any $v_{1}$ as well. Hence, if our constrained problem admits an extremum, this corresponds to the SNIC bifurcation occurring at $(y_{0},v_{1})$ such that

$$\frac{\partial g}{\partial y_{0}}(y_{0},v_{1}) = 0. $$

From (7), we obtain

$$\frac{\partial g}{\partial v_{1}}(y_{0},v_{1}) = - \frac{a}{A} \biggl( \frac {m_{G}^{P}}{m_{G}^{I}}+\frac{B}{b} C_{4} \frac{\partial\hat{\mathcal {S}}}{\partial v}(C_{3} y_{0},v_{0}-v_{1}) \biggr). $$

**Fig. 7:**
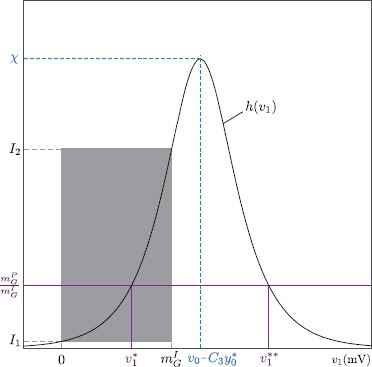
Interval of $m_{G}^{P}/m_{G}^{I}$ values corresponding to a realized local minimum of neural excitability threshold. Graphic representation of the function *h* defined by (19) and interval $[I_{1},I_{2}]$ for which $v_{1}^{*} \in[0,m_{G}^{I}]$

Using the facts that, for any fixed value of $y_{0}$, function $v_{1} \to \frac{\partial g}{\partial v_{1}}(y_{0},v_{1})$ is bell-shaped and its maximum value does not depend on $y_{0}$ (see Fig. 7), one finds that the function $\frac{\partial g}{\partial v_{1}}(y_{0},v_{1})$ vanishes in $v_{1}$ if

14 $$ 0 < \frac{m_{G}^{P}}{m_{G}^{I}} < \chi. $$

If $\frac{m_{G}^{P}}{m_{G}^{I}} \geqslant\chi$, the function $\frac {\partial g}{\partial v_{1}}(y_{0},v_{1})$ admits no zero, which proves the first item of Proposition 4.1.

Now, we assume that condition (14) is fulfilled and we search the values of $v_{1}$ satisfying $\frac{\partial g}{\partial v_{1}}(y_{0},v_{1})=0$, *i.e.*

$$\frac{m_{G}^{P}}{m_{G}^{I}}+\frac{B}{b} C_{4} \frac{\partial\hat{\mathcal {S}}}{\partial v}(C_{3} y_{0},v_{0}-v_{1})=0. $$

Noting $X=e^{r ( v_{0}-v_{1}-C_{3} y_{0} )}=(\mathcal{S}(C_{3} y_{0}, v_{0}-v_{1}, r))^{-1}-1$, this equation reads

15 $$ \frac{m_{G}^{P}}{m_{G}^{I}} X^{2}+2 \biggl(\frac{m_{G}^{P}}{m_{G}^{I}}-2 \chi \biggr) X +\frac{m_{G}^{P}}{m_{G}^{I}}=0. $$

Setting

16 $$ V_{\pm}= 2 \chi \frac{m_{G}^{I}}{m_{G}^{P}}-1\pm2 \sqrt{ \chi \frac {m_{G}^{I}}{m_{G}^{P}} \biggl(\chi \frac{m_{G}^{I}}{m_{G}^{P}}-1 \biggr)}, $$

we obtain the two solutions $v_{1}^{*} < v_{1}^{**}$ of $\frac{\partial g}{\partial v_{1}}(y_{0},v_{1})=0$:

17 $$\begin{aligned} v_{1}^{*} =& v_{0}-C_{3} y_{0}-\frac{1}{r} \ln ( V_{+} ), \end{aligned}$$

18 $$\begin{aligned} v_{1}^{**} =& v_{0}-C_{3} y_{0}-\frac{1}{r} \ln ( V_{-} ). \end{aligned}$$

Note that $v_{1}^{*}$ (resp. $v_{1}^{**}$) corresponds to the extremum when the saddle-node $\mathrm{SN}_{1}$ (resp. $\mathrm{SN}_{2}$) crosses the fold of the surface $g(y_{0},v_{1})=p$. We consider $v_{1}=v_{1}^{*}$ and we note $y_{0}^{*}$ the value of $y_{0}$ corresponding to the SNIC connection for this value of $v_{1}$, *i.e.* the solution of

$$\begin{aligned} \frac{\partial g}{\partial y_{0}}\bigl(y_{0},v_{1}^{*}\bigr) =& 0, \\ \frac{\partial^{2} g}{\partial y_{0}^{2}}\bigl(y_{0},v_{1}^{*}\bigr) < & 0. \end{aligned}$$

To prove that $p_{{\mathrm{SNIC}}}$ reaches a local minimum at $v_{1}=v_{1}^{*}$, we introduce the bordered Hessian matrix *H̅* associated with the Lagrangian function at its singular point $(y_{0},v_{1},\lambda) = (y_{0}^{*},v_{1}^{*},0)$ (solution of system (13a)-(13c)):

$$\begin{aligned} \overline{H}\bigl(y_{0}^{*}, v_{1}^{*},0\bigr) =& \begin{pmatrix} 0 & \frac{\partial^{2} g}{\partial y_{0}^{2}} & \frac{\partial^{2} g}{\partial v_{1}\, \partial y_{0}} \\ \frac{\partial^{2} g}{\partial y_{0}^{2}} & \frac{\partial^{2} \mathsf{L}}{\partial y_{0}^{2}} & \frac{\partial^{2} \mathsf{L}}{\partial v_{1}\, \partial y_{0}} \\ \frac{\partial^{2} g}{\partial v_{1}\, \partial y_{0}} & \frac{\partial^{2} \mathsf{L}}{\partial v_{1}\, \partial y_{0}} & \frac{\partial^{2} \mathsf{L}}{\partial v_{1}^{2}} \end{pmatrix} _{|_{(y_{0}^{*}, v_{1}^{*},0)}} \\ =& \begin{pmatrix} 0 & \frac{\partial^{2} g}{\partial y_{0}^{2}} & \frac{\partial^{2} g}{\partial v_{1}\, \partial y_{0}} \\ \frac{\partial^{2} g}{\partial y_{0}^{2}} & \frac{\partial^{2} g}{\partial y_{0}^{2}} & \frac{\partial^{2} g}{\partial v_{1}\, \partial y_{0}} \\ \frac{\partial^{2} g}{\partial v_{1}\, \partial y_{0}} & \frac{\partial^{2} g}{\partial v_{1}\, \partial y_{0}} & \frac{\partial^{2} g}{\partial v_{1}^{2}} \end{pmatrix} _{|_{(y_{0}^{*}, v_{1}^{*},0)}}. \end{aligned}$$

The determinant of $\overline{H}(y_{0}^{*},v_{1}^{*},0)$ is given by

$$\begin{aligned} &\det{\overline{H}\bigl(y_{0}^{*},v_{1}^{*},0\bigr)} \\ &\quad = - \frac{\partial^{2} g}{\partial y_{0}^{2}}\bigl(y_{0}^{*}, v_{1}^{*}\bigr) \biggl[ \frac{\partial^{2} g}{\partial y_{0}^{2}}\bigl(y_{0}^{*}, v_{1}^{*}\bigr) \frac{\partial^{2} g}{\partial v_{1}^{2}}\bigl(y_{0}^{*}, v_{1}^{*}\bigr)- \biggl( \frac {\partial^{2} g}{\partial v_{1} \, \partial y_{0}}\bigl(y_{0}^{*}, v_{1}^{*}\bigr) \biggr)^{2} \biggr]. \end{aligned}$$

On one hand, the saddle-node associated with the SNIC bifurcation is not degenerate and is a local maximum of $g(y_{0},v_{1})$, thus $\frac {\partial^{2} g}{\partial y_{0}^{2}}(y_{0}^{*}, v_{1}^{*})<0$. On the other hand, for any $y_{0}$, $v_{1} \to\frac{\partial g}{\partial v_{1}}(y_{0},v_{1})$ is increasing at $(y_{0}, v_{1}^{*})$ (see Fig. 7), thus $\frac {\partial^{2} g}{\partial v_{1}^{2}}(y_{0}^{*}, v_{1}^{*})>0$. Finally

$$\det{\overline{H}\bigl(y_{0}^{*},v_{1}^{*},0\bigr)} < 0 $$

and $(y_{0}^{*},v_{1}^{*})$ corresponds to a local minimum of $p_{{\mathrm{SNIC}}}$. A similar argument proves that $(y_{0}^{**},v_{1}^{**})$ corresponds to a local maximum of $p_{{\mathrm{SNIC}}}$ (where $y_{0}^{**}$ is the $y_{0}$ value corresponding to ${{\mathrm{SN}}_{2}}$ bifurcation for $v_{1}=v_{1}^{**}$). □

The above proposition can be interpreted as a necessary condition for having a change in the sense of variations of $p_{{\mathrm{SNIC}}}$ when $v_{1}$ varies in $[0, m_{G}^{I}]$. We now derive a necessary and sufficient condition so that $v_{1}^{*}$ actually lies in $[0, m_{G}^{I}]$. Since $v_{1}^{*}$ satisfies $\frac{\partial g}{\partial v_{1}}(y_{0}^{*},v_{1}^{*})=0$, one obtains, from Eq. (15),

19 $$ \frac{m_{G}^{P}}{m_{G}^{I}} = 4\chi \bigl(1-\mathcal{S} \bigl(C_{3} y_{0}^{*}, v_{0}-v_{1}^{*}, r \bigr) \bigr) \mathcal{S}\bigl(C_{3} y_{0}^{*}, v_{0}-v_{1}^{*}, r\bigr) = 4\chi h\bigl(v_{1}^{*} \bigr). $$

For any $y_{0}$, function $h:v_{1} \mapsto (1-\mathcal{S}(C_{3} y_{0}, v_{0}-v_{1}, r) ) \mathcal{S}(C_{3} y_{0}, v_{0}-v_{1}, r)$ is strictly increasing over $[0,m_{G}^{I}]$, and $0 \leq v_{1}^{*} \leq m_{G}^{I}$ if and only if $I_{1} \leq\frac{m_{G}^{P}}{m_{G}^{I}} \leq I_{2} $ where

20a $$\begin{aligned} I_{1} =& 4\chi \bigl(1-\mathcal{S}\bigl(C_{3} y_{0}^{*}, v_{0}, r\bigr) \bigr) \mathcal {S} \bigl(C_{3} y_{0}^{*}, v_{0}, r\bigr), \end{aligned}$$

20b $$\begin{aligned} I_{2} =& 4\chi \bigl(1-\mathcal{S}\bigl(C_{3} y_{0}^{*}, v_{0}-m_{G}^{I}, r\bigr) \bigr) \mathcal{S}\bigl(C_{3} y_{0}^{*}, v_{0}-m_{G}^{I}, r\bigr). \end{aligned}$$

Figure 7 gives an illustration of the function *h* as well as how $I_{1}$ and $I_{2}$ are built.

In conclusion, for a fixed value of $v_{2}$, $p_{{\mathrm{SNIC}}}$ reaches a local minimum at a value $v_{1}^{*}$ such that $0 \leq v_{1}^{*} \leq m_{G}^{I}$ if and only if

$$\frac{m_{G}^{P}}{m_{G}^{I}} \in (0, \chi ) \cap [I_{1},I_{2} ]. $$

Moreover, in Sect. 3 about the astrocyte GABA uptake deficiency, we proved that, for a fixed value of $v_{1}$, $p_{{\mathrm{SNIC}}}$ is linear and increasing with $v_{2}$. Both results allow us to predict that there exist three shapes of $p_{{\mathrm{SNIC}}}(v_{1},v_{2})$ according to the value of $\frac{m_{G}^{P}}{m_{G}^{I}}$.

- If $\frac{m_{G}^{P}}{m_{G}^{I}}< I_{1}$ then $v_{1}^{*}<0$ and $p_{{\mathrm{SNIC}}}$ strictly increases with $v_{1}$ and $v_{2}$.
- If $\frac{m_{G}^{P}}{m_{G}^{I}}>I_{2}$, then $v_{1}^{*}>m_{G}^{I}$ and $p_{{\mathrm{SNIC}}}$ strictly decreases when $v_{1}$ increases (for $v_{2}$ fixed) and strictly increases with $v_{2}$ (for $v_{1}$ fixed).
- If $I_{1} \leq\frac{m_{G}^{P}}{m_{G}^{I}} \leq I_{2}$, then $0 \leq v_{1}^{*} \leq m_{G}^{I}$ and $p_{{\mathrm{SNIC}}}$ decreases when $v_{1}$ increases in $[0,v_{1}^{*}]$ and increases with $v_{1}>v_{1}^{*}$ (for $v_{2}$ fixed).

In the following we illustrate the three qualitative types of neural activity resulting from an astrocyte deficiency to capture glutamate using the following values:

$$\begin{aligned} (\mathrm{a})&\quad \frac{m_{G}^{P}}{m_{G}^{I}} = 1.7 < I_{1}, \\ (\mathrm{b})&\quad \frac{m_{G}^{P}}{m_{G}^{I}} = 3.2 >I_{2}, \\ (\mathrm{c})&\quad \frac{m_{G}^{P}}{m_{G}^{I}} = 2.43 \in [I_{1},I_{2} ]. \end{aligned}$$

**Fig. 8:**
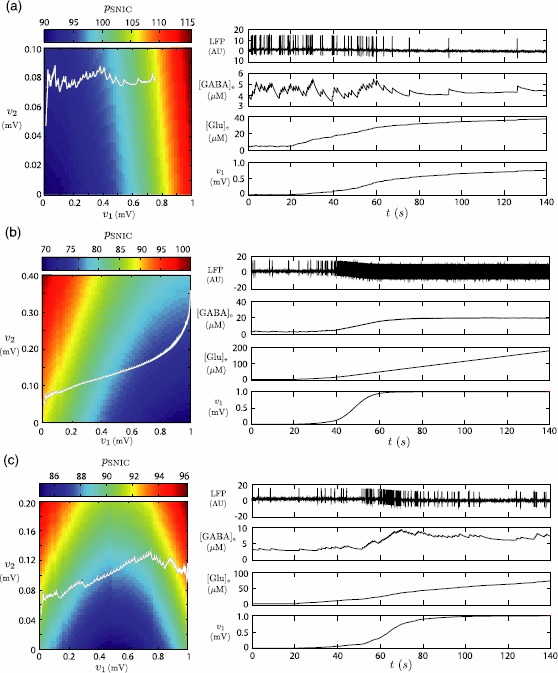
Alteration of astrocytic glutamate uptake: (**a**) lessening the excitability, (**b**) resulting in sustained hyperexcitability, (**c**) resulting in transient hyperexcitability. In each case, *the colormap on the left* displays the value of $p_{{\mathrm{SNIC}}}$ in $(v_{1},v_{2})$ plane, and the time series on *the right* correspond to LFP, ${[\mathrm{GABA}]_{\mathrm{e}}}$, ${[\mathrm{Glu}]_{\mathrm{e}}}$ and $v_{1}=m_{G}^{I} {\mathcal{S}} ({[\mathrm{Glu}]_{\mathrm{e}}}, v_{G}, r_{G})$. *The black curves on the colormap* are the trace of ($m_{G}^{I} {\mathcal{S}}({[\mathrm{Glu}]_{\mathrm{e}}}, v_{G}, r_{G})$, $m_{\gamma } {\mathcal{S}}({[\mathrm{GABA}]_{\mathrm{e}}}, v_{\gamma}, r_{\gamma})$) along the associated orbits of the model. At $t=20~\mbox{s}$, we alter the glutamate astrocytic uptake by setting $V_{G}^{{\mathrm{ae}}}=0$. The three cases are obtained with: (a) $\frac{m_{G}^{P}}{m_{G}^{I}}=1.7$, (b) $\frac {m_{G}^{P}}{m_{G}^{I}}=3.2$, (c) $\frac{m_{G}^{P}}{m_{G}^{I}}=2.43$. All other parameters are the same in the three cases and given in Table 1

For each case, we provide simulations representing the value of $p_{{\mathrm{SNIC}}}$ in $(v_{1},v_{2})$ space and time series generated by the model when the glutamate astrocytic uptake is altered (Fig. 8).

For $\frac{m_{G}^{P}}{m_{G}^{I}}=1.7< I_{1}$ (panel (a) of Fig. 8), $v_{1}^{*}$ is negative and thus $p_{{\mathrm{SNIC}}}$ increases with $v_{1}$. Hence in this scenario, when $v_{1}$ increases as a consequence of the astrocytic glutamate uptake deficiency, we expect to observe a decrease in the oscillation frequency in the neural activity. This is possible because, although a reduction of astrocytic glutamate uptake is paired with a strong decrease of neural activity, it limits synaptic glutamate release allowing deficient astrocytic uptake to still stabilize extracellular glutamate levels and thus neural activity.

Panel (b) of Fig. 8 is obtained with $\frac {m_{G}^{P}}{m_{G}^{I}}=3.2>I_{2}$. Since $v_{1}^{*}>1$, $p_{{\mathrm{SNIC}}}$ decreases as $v_{1}$ increases. Thereby, an astrocyte glutamate uptake deficiency triggers an increase of the $v_{1}$ value, and we observe an increase in the oscillation frequencies in the neural time series. Since the astrocytic uptake is reduced, glutamate accumulates in the extracellular space and the corresponding concentration baseline increases drastically.

Finally, the intermediate scenario where $\frac {m_{G}^{P}}{m_{G}^{I}}=2.43\in[I_{1},I_{2}]$ is shown in panel (c) of Fig. 8. In this case $p_{{\mathrm{SNIC}}}$ decreases for $v_{1}< v_{1}^{*}$ and increases otherwise. Therefore, in the presence of deficient astrocytic glutamate uptake, we observe only a transient increase of the frequency of LFP spikes. This is the scenario presciently mentioned at the beginning of the section where the effect of increased extracellular glutamate levels on neuronal excitability are counterbalanced by increased extracellular GABA levels resulting from increased interneurons firing and accounts for the delay observed in our simulations in reaching steady frequency of LFP oscillations.

This scenario is physiologically relevant insofar as it is conceivable that an excess of extracellular glutamate concentration is regulated after a delay, triggering a decrease of neural activity after the initial increase. Moreover, the frequency after the regulation delay can be greater or lower than the initial one, depending on the value of the ratio $\frac{m_{G}^{P}}{m_{G}^{I}}$. Note that this value can be tuned to obtain $v_{1}^{*}$ small enough and $p_{{\mathrm{SNIC}}}$ large enough so that the frequency after regulation is equal or lower than the one before uptake deficiency. This property offers the possibility of fitting the model outputs to experimental data and allows us to propose hypotheses about physiological and pathological mechanisms.

## Discussion and Conclusions

We have introduced a novel neuron–astrocyte mass model, which combines two previously studied models [29, 30] by bidirectional neuron–astrocyte coupling based on astrocytic uptake-mediated modulations of extracellular glutamate and GABA concentrations and their effect on neural activity in agreement with experimental observations [10, 37, 44, 45].

Using analytical arguments of bifurcation theory and contained optimization, we have characterized different types of change in the neural activity behavior under the impact of the neurotransmitters-mediated modulation of neuron excitability. Based on the interpretation of the aggregated—yet biophysically significant parameters of our model—we have reproduced *in silico* both glutamate and GABA uptake deficiency in astrocytes and illustrated by numerical simulations the different types of change in the neural activity resulting from such deficiency.

Our study leads to several predictions. First is the observation that deficiency of GABA uptake by astrocytes increases the threshold for neuronal activation in a linear fashion. In other words, neurons tends to fire more the slower astrocyte GABA uptake is, or equivalently, the larger extracellular GABA concentration is, which is consistent with experimental findings [46, 47]. The second prediction comes instead from the analysis of the neuronal response in the presence of deficient astrocytic glutamate uptake. In this case, neural activity may either be reduced or enhanced or, alternatively, may display a transient of high activity before stabilizing around a new regime whose firing frequency is close to the one measured in the absence of astrocyte deficiency. A prominent feature of our model is its mathematical tractability, despite the fact that, differently from the sole neural mass model in [29], an explicit formula for the SNIC bifurcation underpinning the rise of neural activity in the presence of bilateral neuron–astrocyte coupling cannot be obtained. It is nonetheless possible to recast the characterization of variations of neuronal excitability upon astrocytic uptake as an optimization problem under an equality constraint resulting from the implicit characterization of the SNIC bifurcation. In particular, we identify by the quantity $m_{G}^{P}/m_{G}^{I}$, which denotes the ratio between maximally affecting concentrations of extracellular glutamate impacting on excitability of pyramidal neurons and interneurons, respectively, the biophysical conditions for the occurrence of different neural activity regimes caused by deficient astrocytic glutamate uptake. In this regard, our analysis suggests that a neural population may counteract the effect of excess extracellular glutamate resulting from deficient astrocytic uptake only if the value of $\frac {m_{G}^{P}}{m_{G}^{I}}$ lies in a well-defined interval $[I_{1},I_{2}]$ which depends on inherent biophysical and cellular properties of the population under consideration. Outside this interval, for values of $\frac{m_{G}^{P}}{m_{G}^{I}} < I_{1}$, the frequency of neural activity decreases and may enter quiescence while for values of $\frac {m_{G}^{P}}{m_{G}^{I}} > I_{2}$, neural firing drastically and persistently increases leading to epileptiform activity in agreement with experiment [48]. On the other hand, for $I_{1} < \frac {m_{G}^{P}}{m_{G}^{I}} < I_{2}$ neural activity may recover to close-to-baseline levels after a high frequency transient, regardless of large extracellular concentrations of glutamate and GABA. We note that while both $I_{1}$ and $I_{2}$ depend on the strength of inhibition of pyramidal neurons by interneurons only $I_{2}$ further depends on $m_{G}^{I}$. Hence, one might interpret the latter dependence as an estimator of the balance between glutamatergic astrocytic feedbacks on pyramidal neurons and correlated interneuron hyperexcitability that the system should seek to recover from astrocytic uptake deficits and avoid hyperexcitability.

A further interesting prediction of our model is that, when $\frac {m_{G}^{P}}{m_{G}^{I}} > I_{1}$, an increase of extracellular glutamate due to deficits in astrocytic uptake develops in parallel with an increase of extracellular GABA which may even be larger than the one expected in an alternative scenario of faulty GABAergic uptake by astrocytes. Dynamically, this GABA increase is necessary for the system to reach a steady low-frequency activity but it may not be sufficient to properly counterbalance the positive feedback on neuronal activation exerted by the faulty astrocytic glutamate uptake. This suggests GABA increases induced by excess extracellular glutamate and the related neuronal activity as a putative biophysical correlate to distinguish between different mechanisms for rise of neural hyperexcitability. In light of the current technological limits whereby monitoring of extracellular neurotransmitters dynamics in humans is not available, these results could have important translational implications, helping to design either new experimental protocols to characterize and identify epileptogenic foci as well as to define more accurate treatments of neuropathogenic states associated with neuronal hyperexcitability or lack thereof.
